# Supplementary material for: Perinatal inflammation influences but does not arrest rapid immune development in preterm babies
Source: Nat Commun. 2020 Mar 9;11:1284. doi: 10.1038/s41467-020-14923-8 (PMC7062833; doi:10.1038/s41467-020-14923-8)
Supplement: Supplementary file 1 — Supplementary Information [file 41467_2020_14923_MOESM1_ESM.pdf]

Supplementary Information

Perinatal Inflammation Influences but Does Not Arrest Rapid Immune Development in Preterm Babies

Kamdar et al.

Deviation of the neonatal immune profile from adult levels

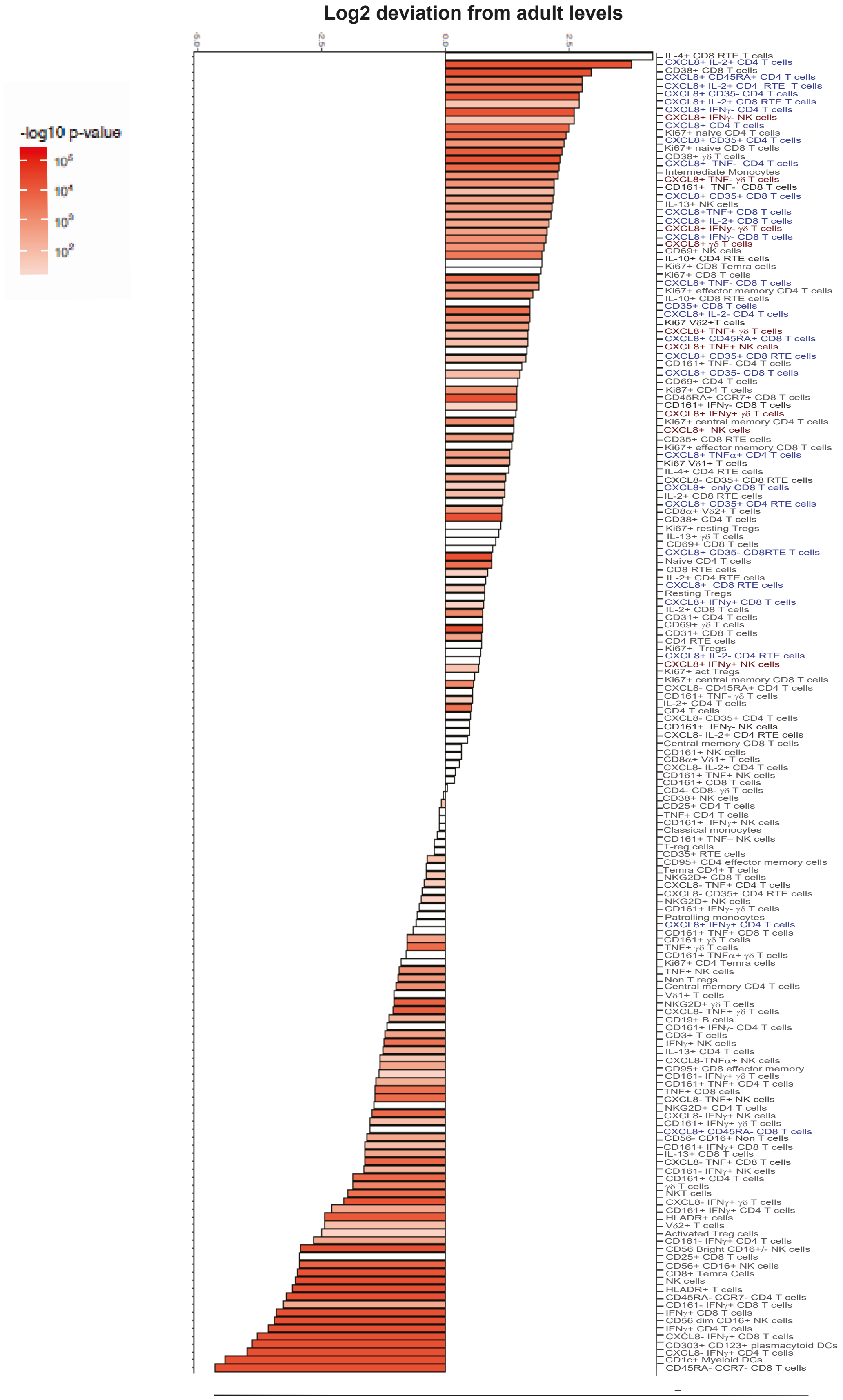

Supplementary Figure 1: Comparison of neonatal and adult immune parameters
Longitudinal PBMC samples from 39 preterm babies were phenotyped for 166 different immune populations by flow cytometry following surface and intracellular staining. For cytokine detection, samples were activated in vitro with PI (4 h, in the presence of BFA) prior to staining. (A) Bar chart depicting the deviation of the neonatal immune profile from adult levels. Plot represents Log2 fold difference of mean neonatal immune cell subset frequencies (n=39 infants) relative to mean adult levels (n=9 adults). Colour indicates Benjamini-Hochberg corrected p values from two tailed nonparametric Wilcoxon rank sum test. Populations highlighted in blue text indicate adaptive CXCL8+ cells and those highlighted in red indicate innate (NK and $\gamma\delta$) CXCL8+ cells.Source data are provided as a Source Data file

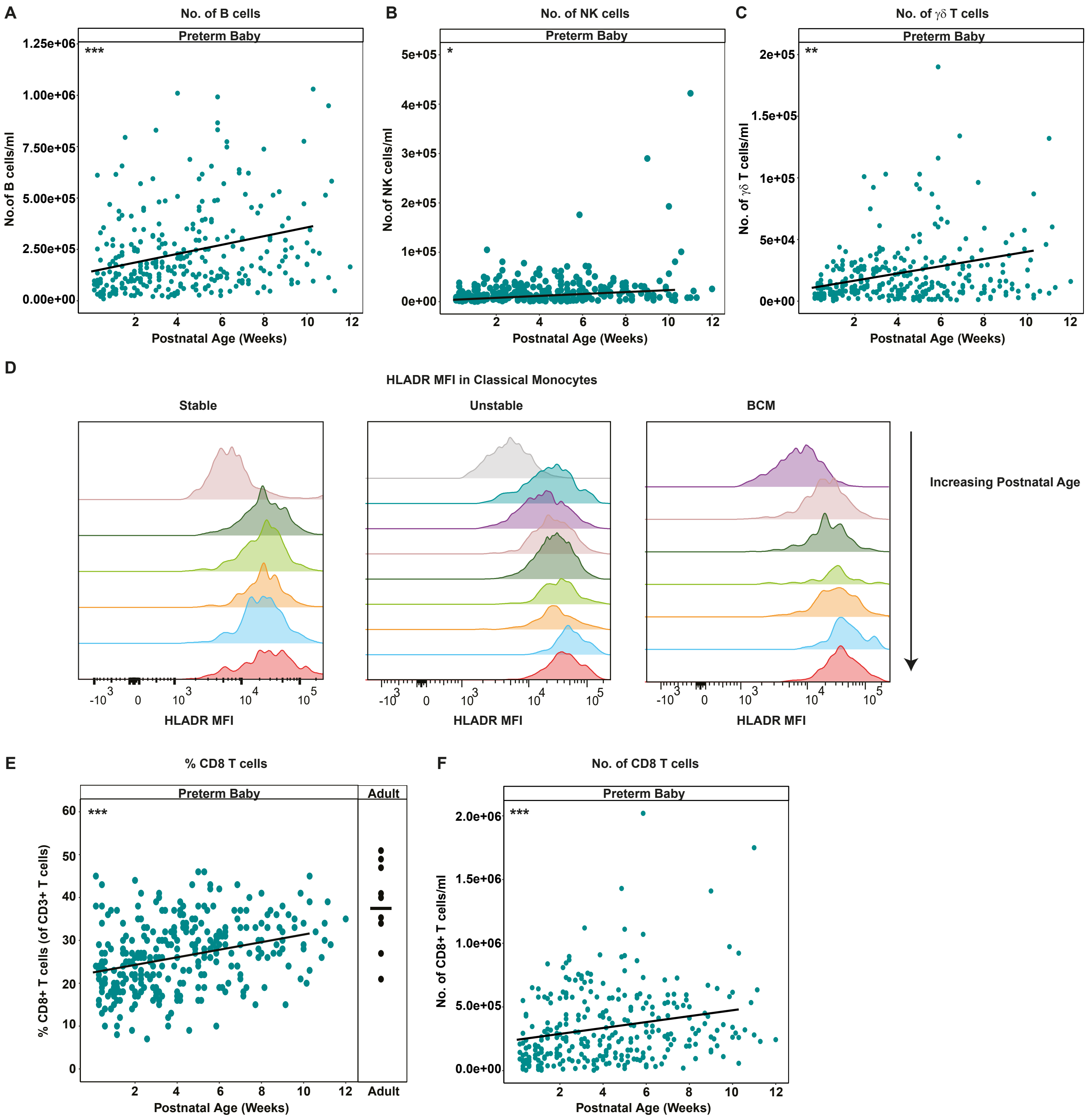

Supplementary Figure 2: Immune parameters that increase with age

The mean fluorescence intensity of HLADR expression in classical monocytes and the frequencies of CD19+ B cells, CD8 T cells, NK cells and NKG2D+ NK cells were examined in longitudinal PBMC samples from 39 preterm babies by flow cytometry. Scatter plot depicting actual numbers/ml of (A) CD19+ B cells. (B) NK cells and (C) γδ T cells. (D) Representative histograms depicting HLADR MFI in classical monocytes with increasing postnatal age in a Stable (left panel), Unstable (middle panel) and BCM (right panel) baby. Scatter plots depicting frequecies of (E) CD8 T cells in preterm babies and adults and actual numbers/ml of (F) CD8 T cells in preterm babies as a function of postnatal age. Data shown in Figures (A-C) and (E-F) are a pool of longitudinal samples from 39 preterm babies where each circle represents an individual sample. *** $p< 0.001$, ** $p < 0.01$ and * $p< 0.05$ as determined by linear mixed effect modelling using the lmer package in R. Source data are provided as a Source Data file.

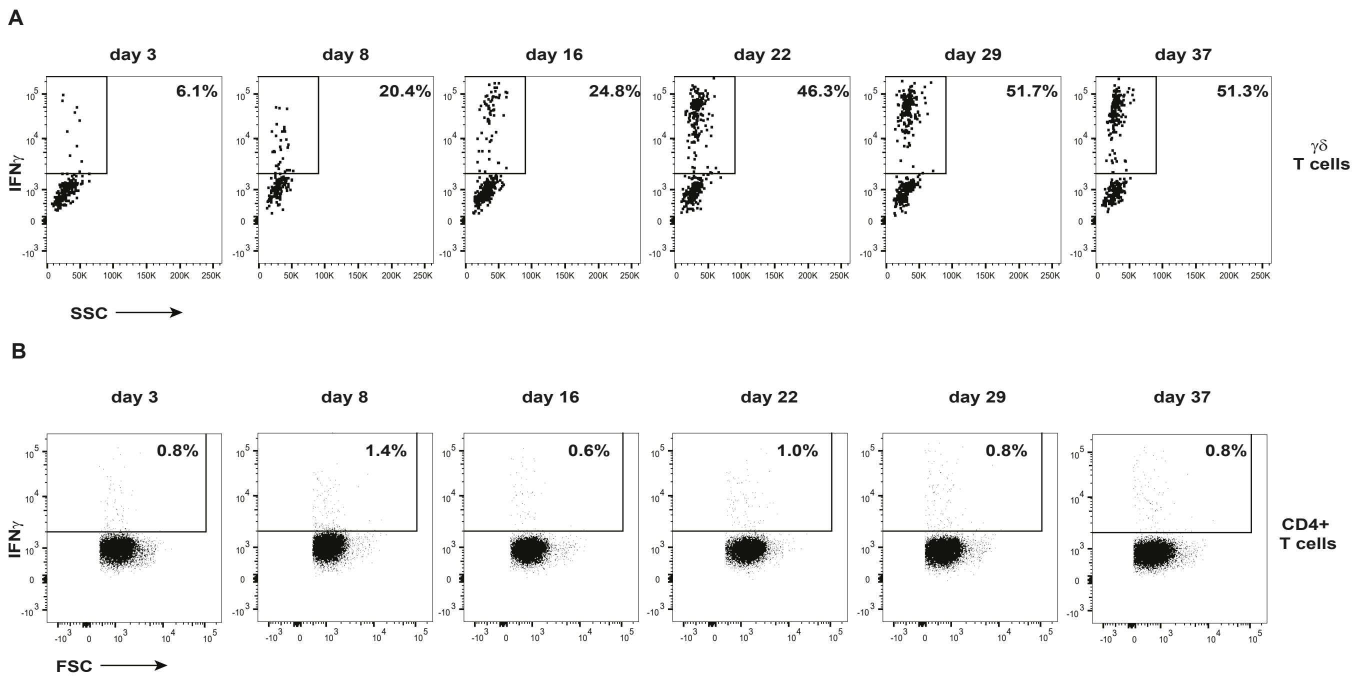

Supplementary Figure 3: IFN-γ expression in γδ and CD4 T cells

Longitudinal PBMC samples from 39 preterm babies were activated in vitro with PI (4 h, in the presence of BFA) and expression of IFN-γ in γδ or CD4 T cells assessed by flow cytometry. Dot plots showing representative staining of IFN-γ expression by (A) γδ T cells and (B) CD4+ T cells in longitudinal samples from a single preterm baby. The postnatal age of the baby at the time the sample was taken is indicated above each dot plot.

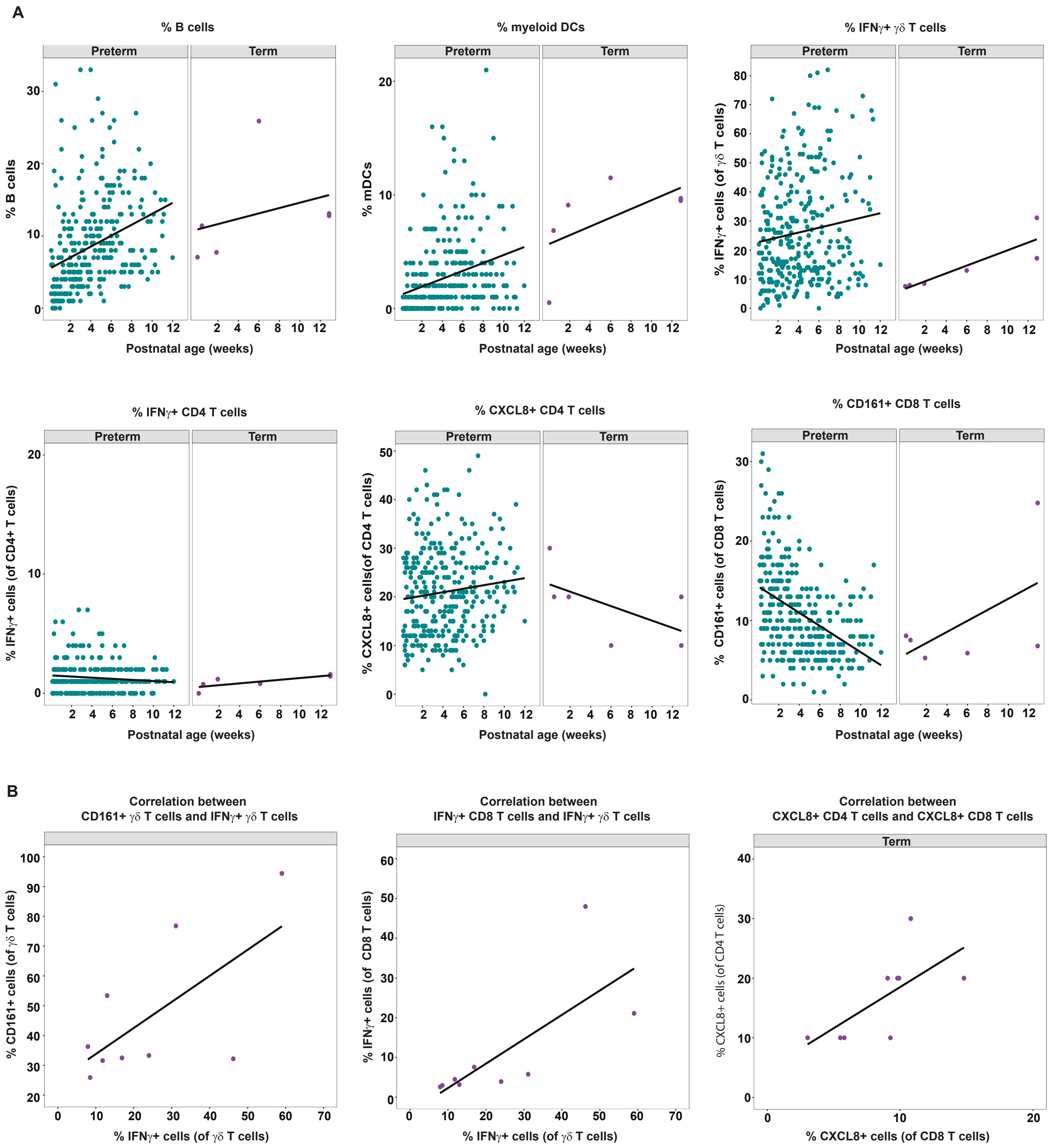

Supplementary Figure 4: Immune development in term infants.
PBMC samples were taken with informed consent from term infants prior to cardiac surgery and were phenotyped for different immune populations by flow cytometry following surface and intracellular staining. For cytokine detection, samples were activated in vitro with PI (4 h, in the presence of BFA) prior to staining (A) Changes in individual immune parameters over time depicted by scatter plots showing frequencies in different parameters (as indicated) in preterm babies (left panel;cyan circles) as a function of postnatal age compared to different term infants sampled at ages up to 3 months (right panel; purple circles). (B) Plots depicting correlation between frequency of (left panel) IFN-γ expressing gd T cells and CD161-expressing γδ T cells; (middle panel) IFN-γ+ CD8 T cells and IFN-γ+ γδ T cells; and (right panel) CXCL8-producing CD4 and CD8 T cells in different term infants (n=9) sampled at different ages within the first 6 months of life.Source data are provided as a Source Data file.

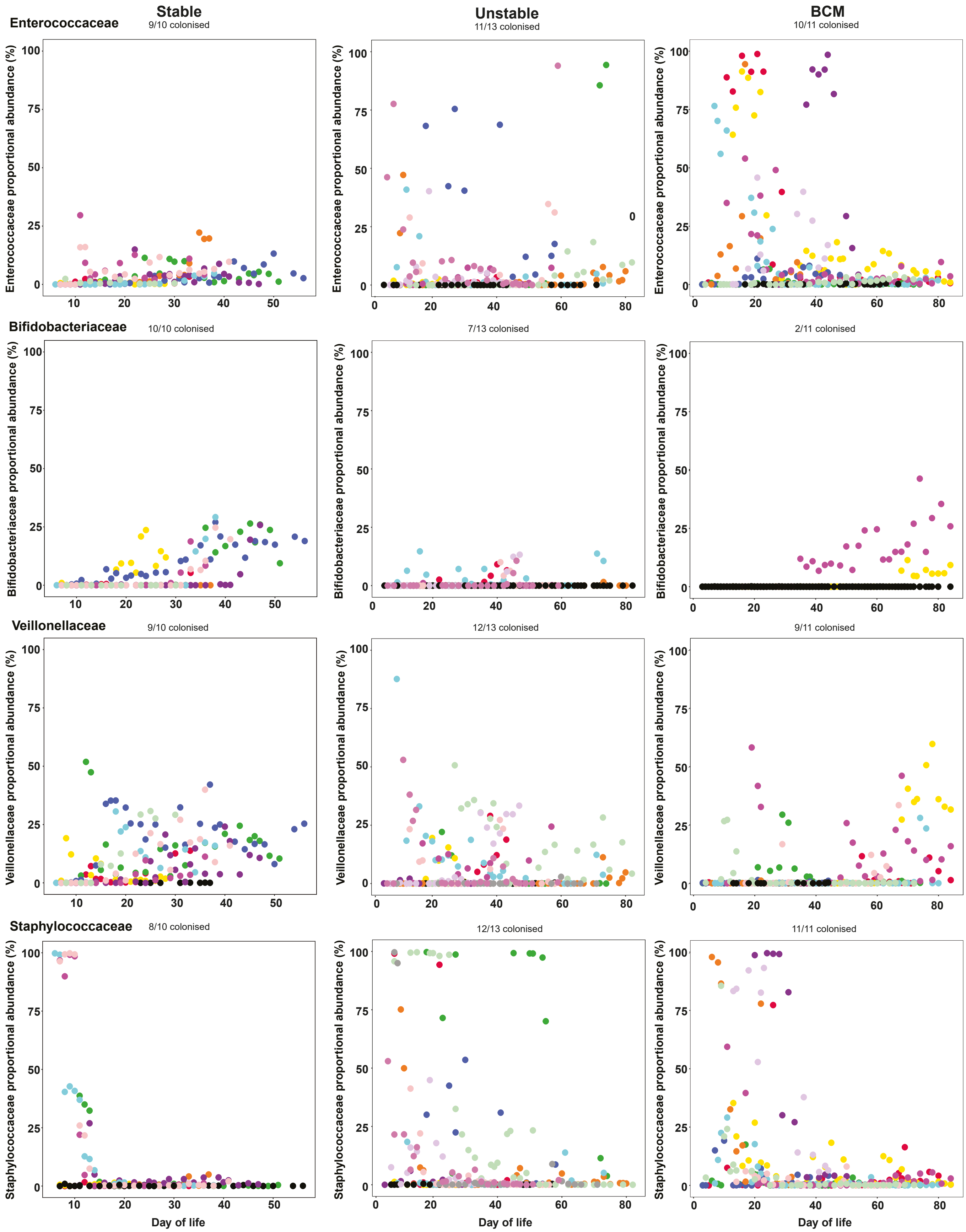

Supplementary Figure 5: Microbiome development

Faecal samples (713) were longitudinally collected from 34 babies across the duration of the study. Bacterial DNA was extracted, and the 16S rRNA gene (hypervariable region V4) was amplified, sequenced and compared against the sequence database to ascertain the relative abundance of bacterial taxa in samples. We have utilised our own curated database, created to focus upon species previously encountered in preterm intestinal microbiome studies. Mean abundance of minority taxa was plotted against time for all samples and participants. Dot plots represent microbiome development over time (as indicated) in individual samples from Stable (left panel), Unstable (middle panel) and BCM babies (right panel). Symbol colours represent samples originating from same baby; Babies who were never colonised with that taxon (i.e. relative abundance always <1%) are uniformly coloured in black. Source data are provided as a Source Data file.

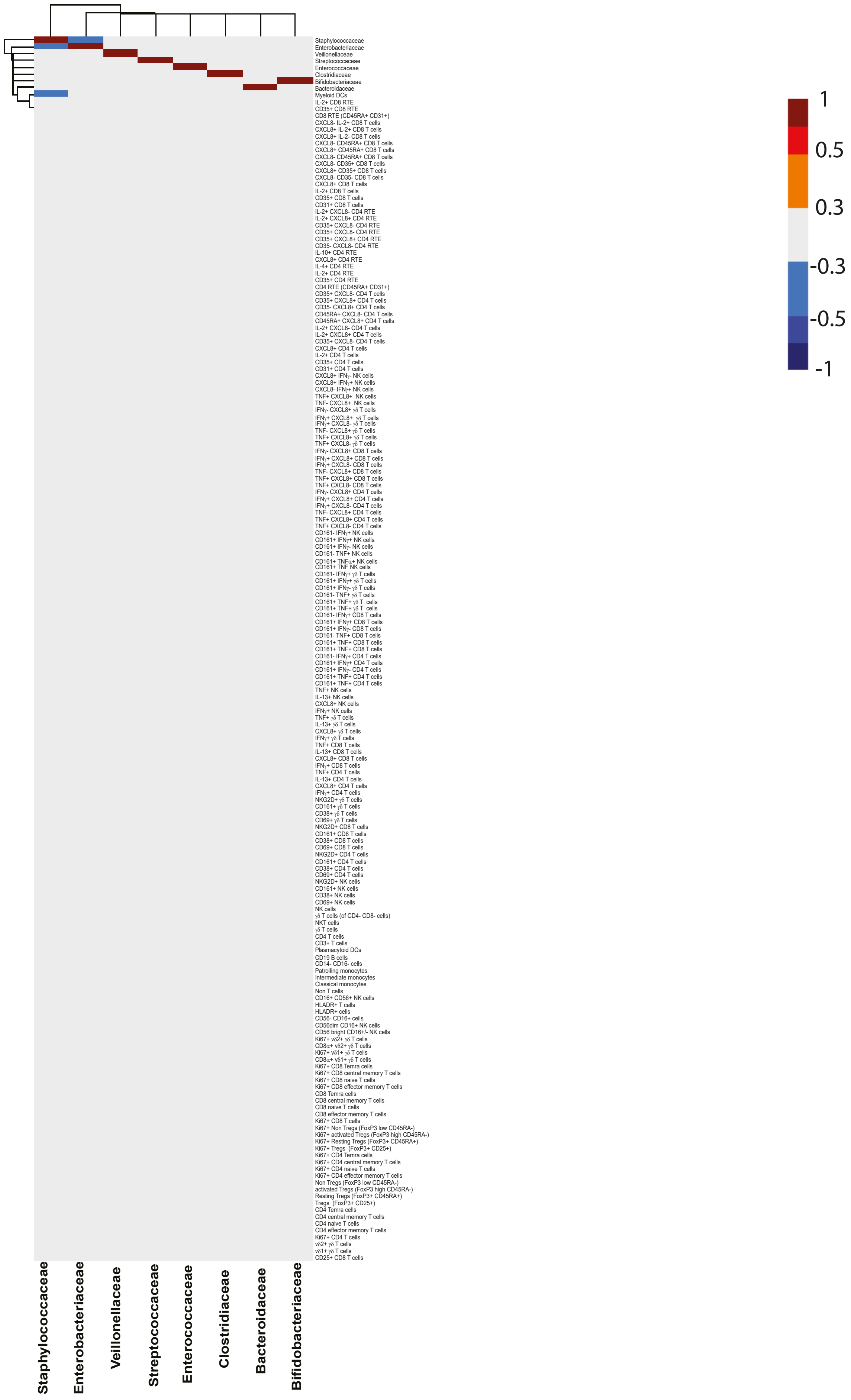

Supplementary Figure 6: Immune microbiome correlations

Heat map depicting aggregate intra-individual spearman correlations between microbiome parameters and immune parameters. Colour indicates R value, only correlations with statistically significant R values of <-0.3 or >0.3 are plotted. Samples from 27 babies were included in this analysis to ensure > 4 samples were included per baby.Source data are provided as a Source Data file.

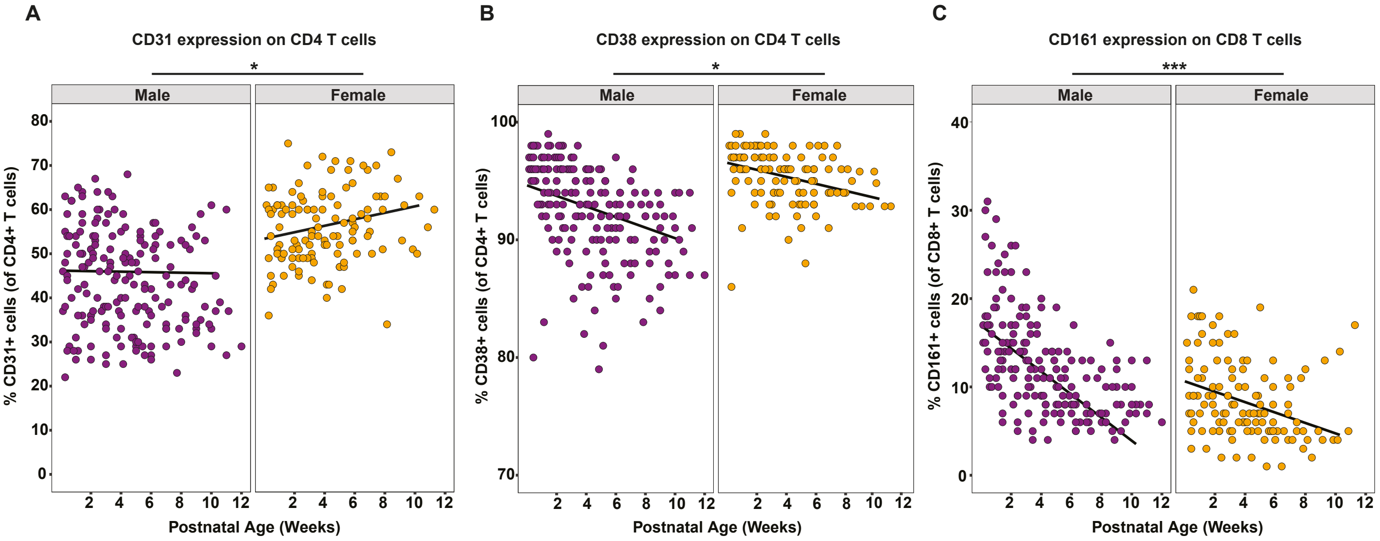

Supplementary Figure 7: Sexual dimorphism in immune parameters

Surface expression of CD31, CD38 on CD4 T cells and CD161 on CD8 T cells was examined by flow cytometry in longitudinal PBMC samples from 39 preterm babies. Compared to males, female babies express significantly higher levels of CD31 and CD38 on CD4+ T cells and significantly lower levels of CD161 on CD8+ T cells as depicted by scatter plots showing frequencies of (A) CD31 and (B) CD38 in CD4 T cells and (C) CD161 on CD8+ T cells in males (left panel, n=23 babies with mean of 8 samples/baby) compared to females (right panel, n=16 with mean of 8 samples/baby) as a function of postnatal age. Data shown are a pool of longitudinal samples from 39 preterm babies where each circle represents an individual sample. *** $p < 0.001$, **$p < 0.01$ and *$p < 0.05$ as determined by linear mixed effect modelling using the lmer package in R.Source data are provided as a Source Data file.

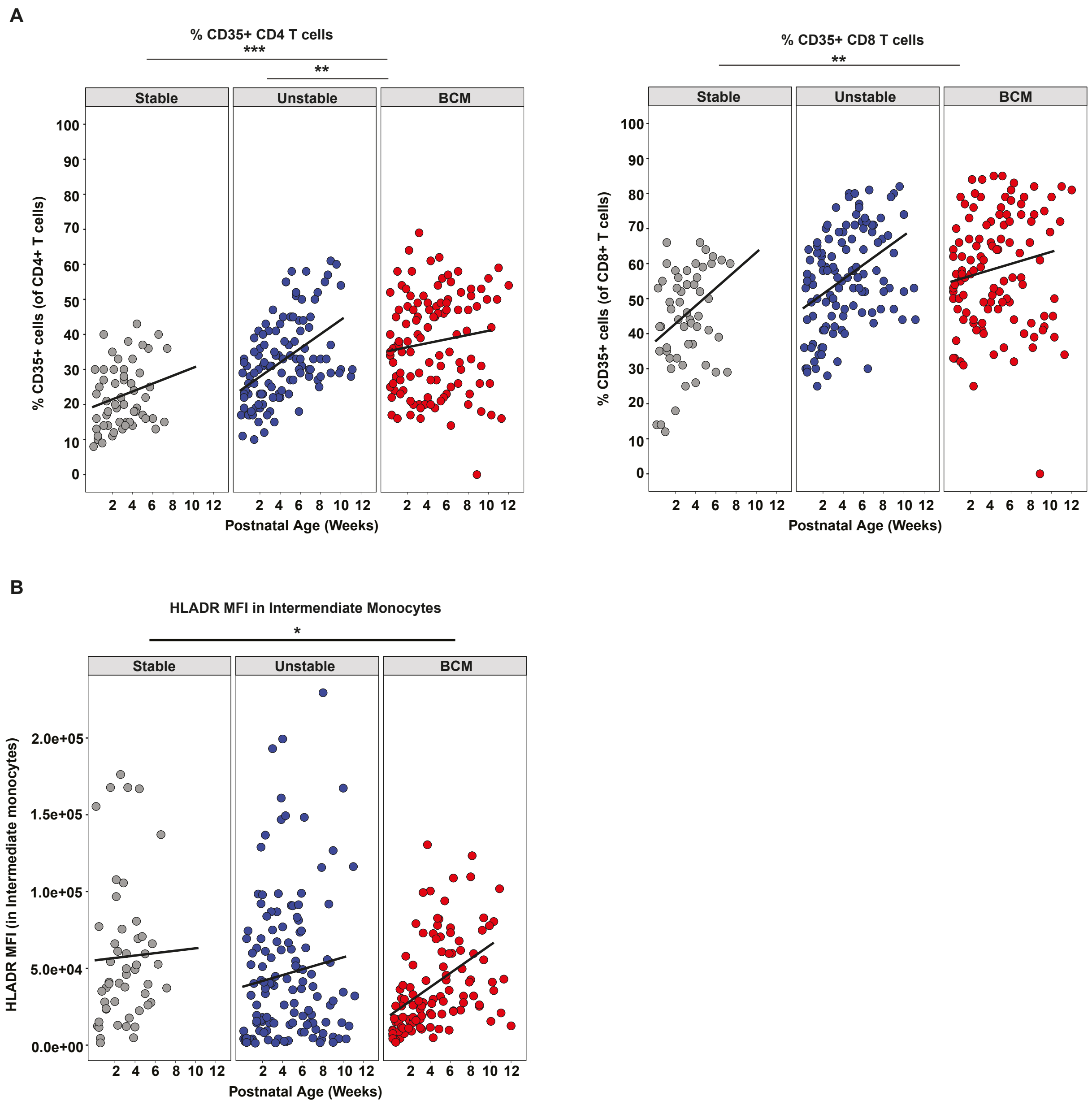

Supplementary Figure 8: CD35 expression in CD4 and CD8 T cells and HLADR MFI in intermediate monocytes

The frequency of CD35-expressing CD4 and CD8 T cells and HLADR MFI in monocytes was examined by flow cytometry in longitudinal PBMC samples from 39 preterm babies. Scatter plots depicting the frequency/MFI (as indicated) of (A) CD35+ CD4+ T cells, (B) CD35+ CD8+ T cells and (C) HLADR MFI in intermediate monocytes in Stable babies (left panel; grey circles, n=10), Unstable babies (middle panel; blue circles, n=16) and BCM babies (right panel; red circles, n=13) as a function of postnatal age. Data shown are a pool of longitudinal samples from 39 preterm babies where each circle represents an individual sample and on average there are 8 longitudinal samples/baby. *** p< 0.001, **p < 0.01 and *p< 0.05 as determined by linear mixed effect modelling using the lmer package in R.Source data are provided as a Source Data file.

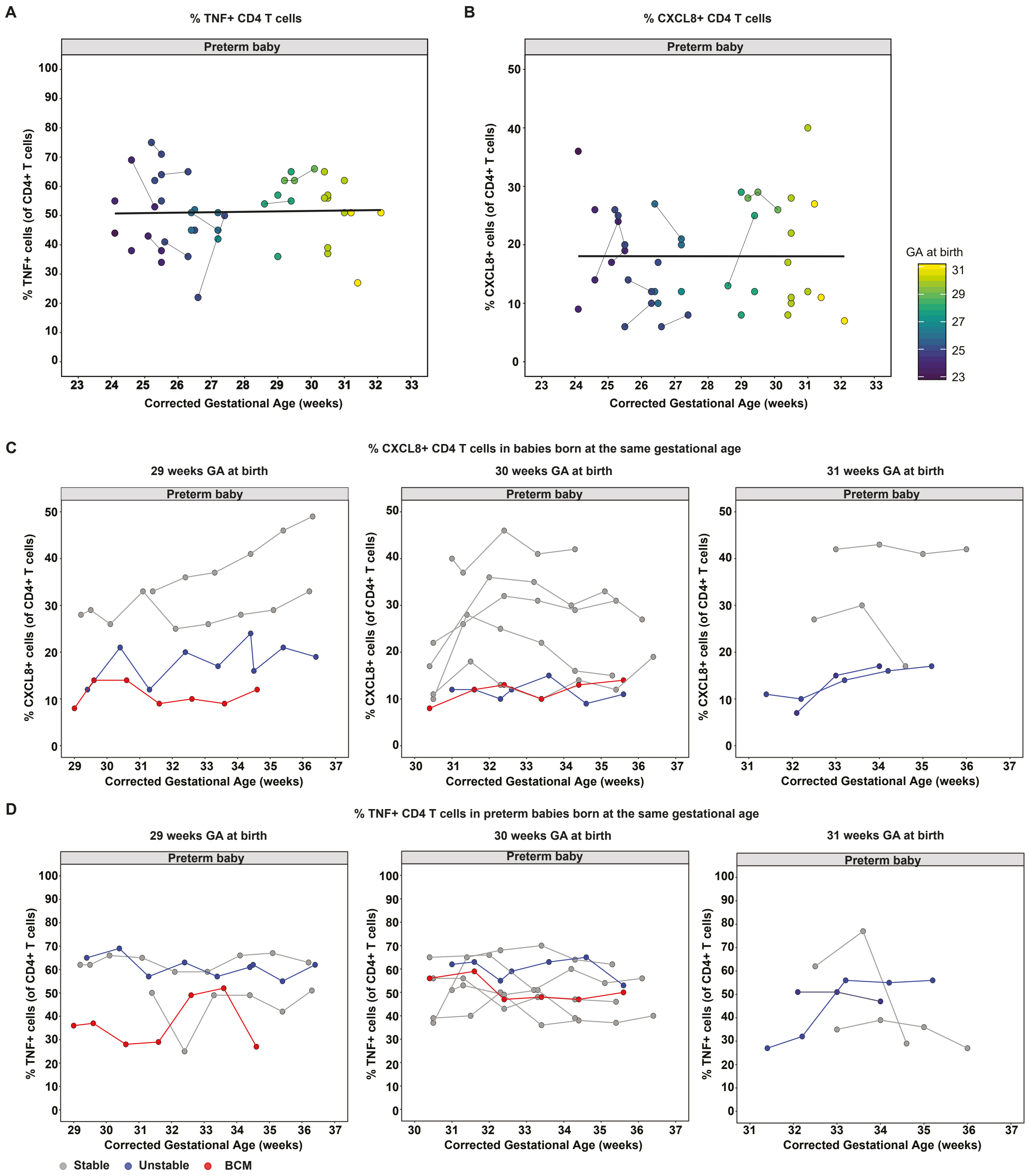

Supplementary Figure 9: T cell expression of CXCL8 or TNF is not dependent on gestational age at birth
Longitudinal PBMC samples from 39 preterm babies were activated in vitro with PI (4 h, in the presence of BFA) and expression of CXCL8 and TNF in CD4+ T cells was assessed by flow cytometry. Scatter plots show frequencies of CD4 T cells expressing (A) TNF and (B) CXCL8 in preterm babies as a function of corrected gestational age (cGA). Data shown are a pool of samples taken in the 1st week of life from 39 preterm babies where each circle represents an individual sample. Linked samples indicate consecutive samples from the same baby within the 1st week of birth. Circles are coloured based on the gestational age (GA) of the baby at birth. The colour gradient is from dark (babies born at earlier GA) to light (babies born at later GA). Unstable and BCM babies born at the GA as stable babies consistently express lower frequencies of CXCL8 but not TNF as depicted by scatter plots showing frequencies of CD4+ T cells expressing: (C) CXCL8 and (D) TNF as a function of cGA in babies born at 29 weeks cGA (left panel), 30 weeks cGA (middle panel) and 31 weeks cGA (right panel). In figures C-D, circles depict individual samples from each baby. Linked samples are longitudinal samples from the same baby. Grey circles represents Stable babies, blue circles represent Unstable babies and Red circles represent BCM babies. *** p< 0.001, **p < 0.01 and *p< 0.05 as determined by linear mixed effect modelling using the lmer package in R.Source data are provided as a Source Data file.

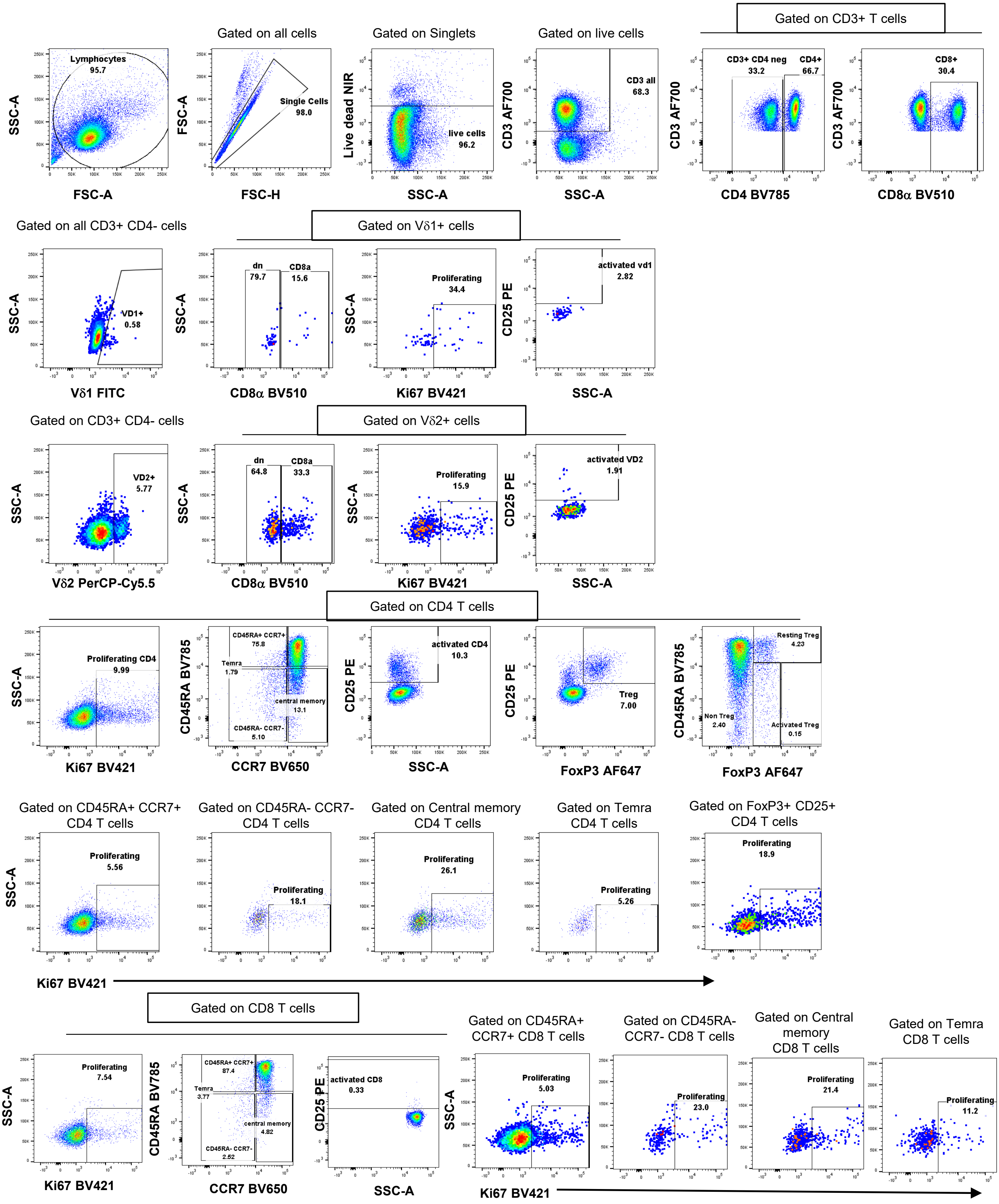

Supplementary figure 10: Gating strategy used to Treg subsets, naïve, effector and memory subsets in CD4 and CD8 T cells, Ki67 and CD25 expression in CD4, CD8 and Vδ1 and Vδ2 T cells and CD8α expression in Vδ1 and Vδ2 T cells by flow cytometry. This strategy was used to examine relevant parameters shown in figures 3 and 5 and supplementary figures 1 and 6

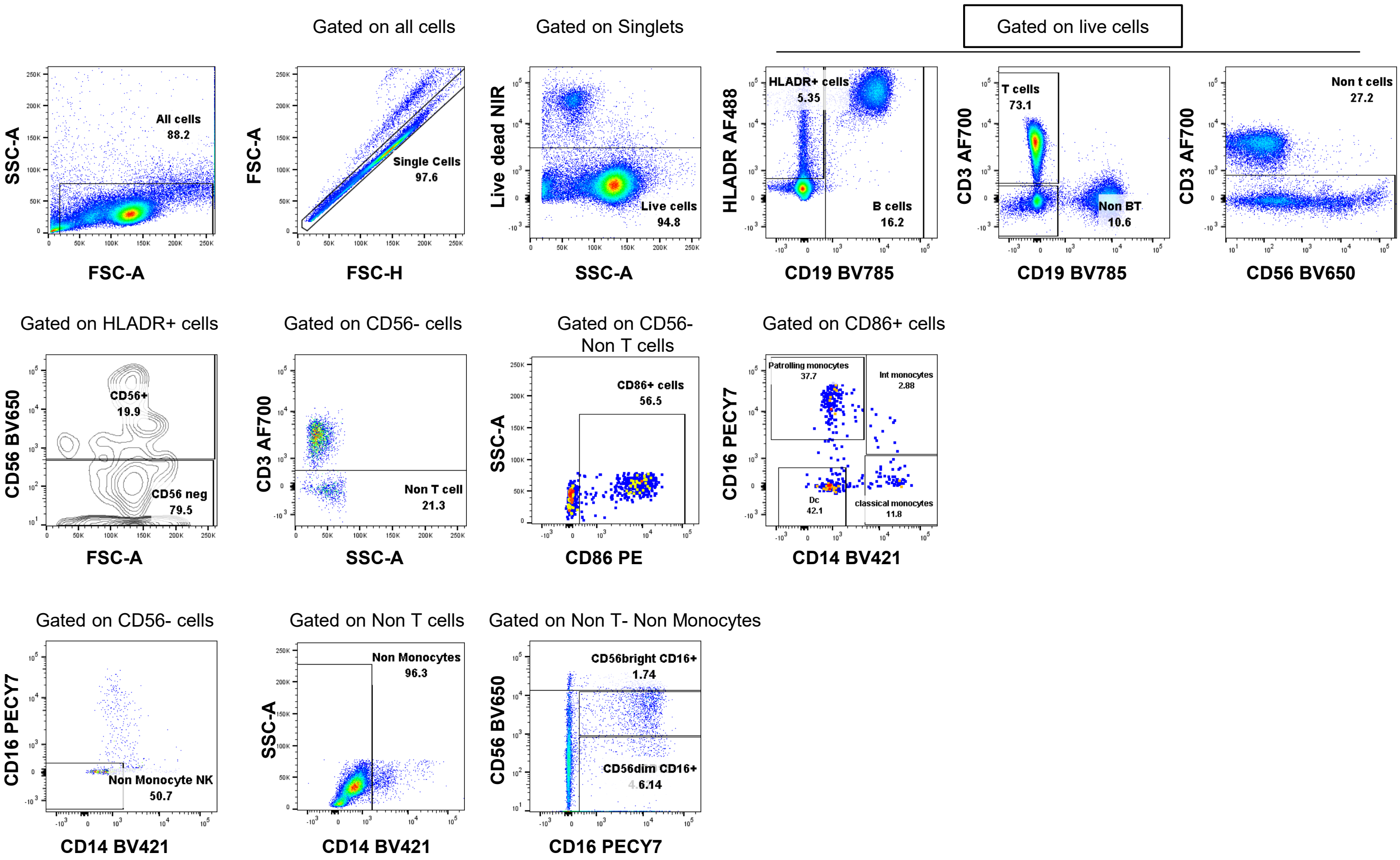

Supplementary figure 11: Gating strategy used to examine proportions of monocytes and CD56/CD16 expressing NK cells by flow cytometry. This strategy was used to examine relevant parameters shown in figures 2,3 and 5 and supplementary figures 1,2,8 and 6

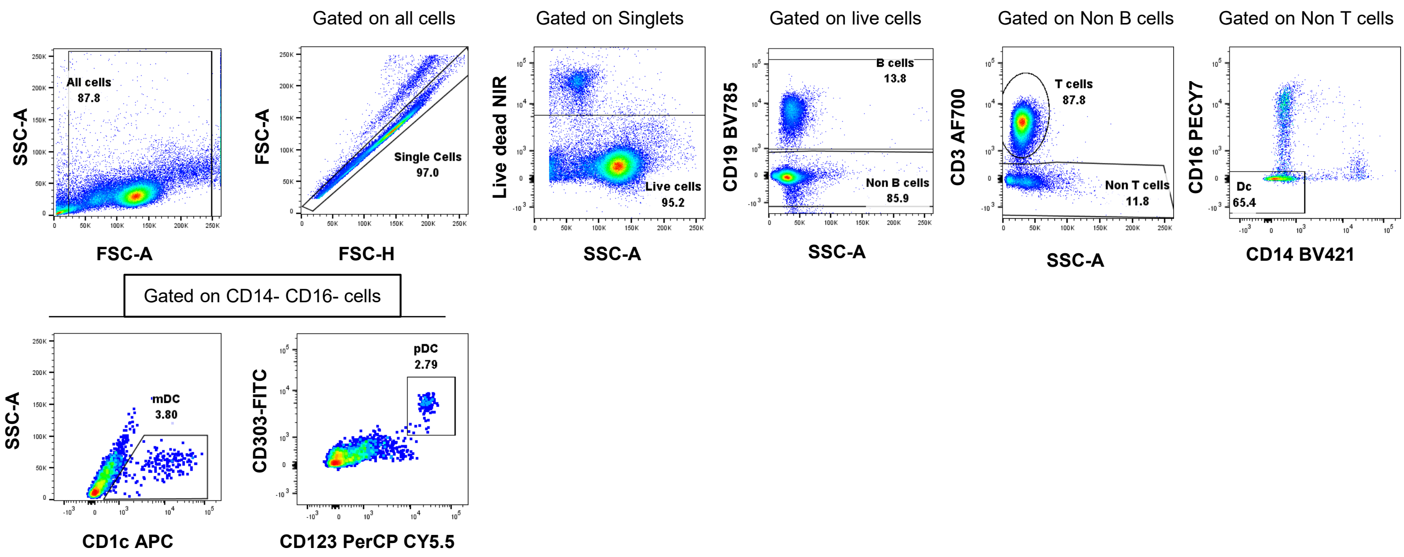

Supplementary figure 12: Gating strategy used to examine proportions of B cells, myeloid and plasmacytoid dendritic cells by flow cytometry. This strategy was used to examine relevant parameters shown in figures 2, 3 and 5 and supplementary figures 1,2,4 and 6

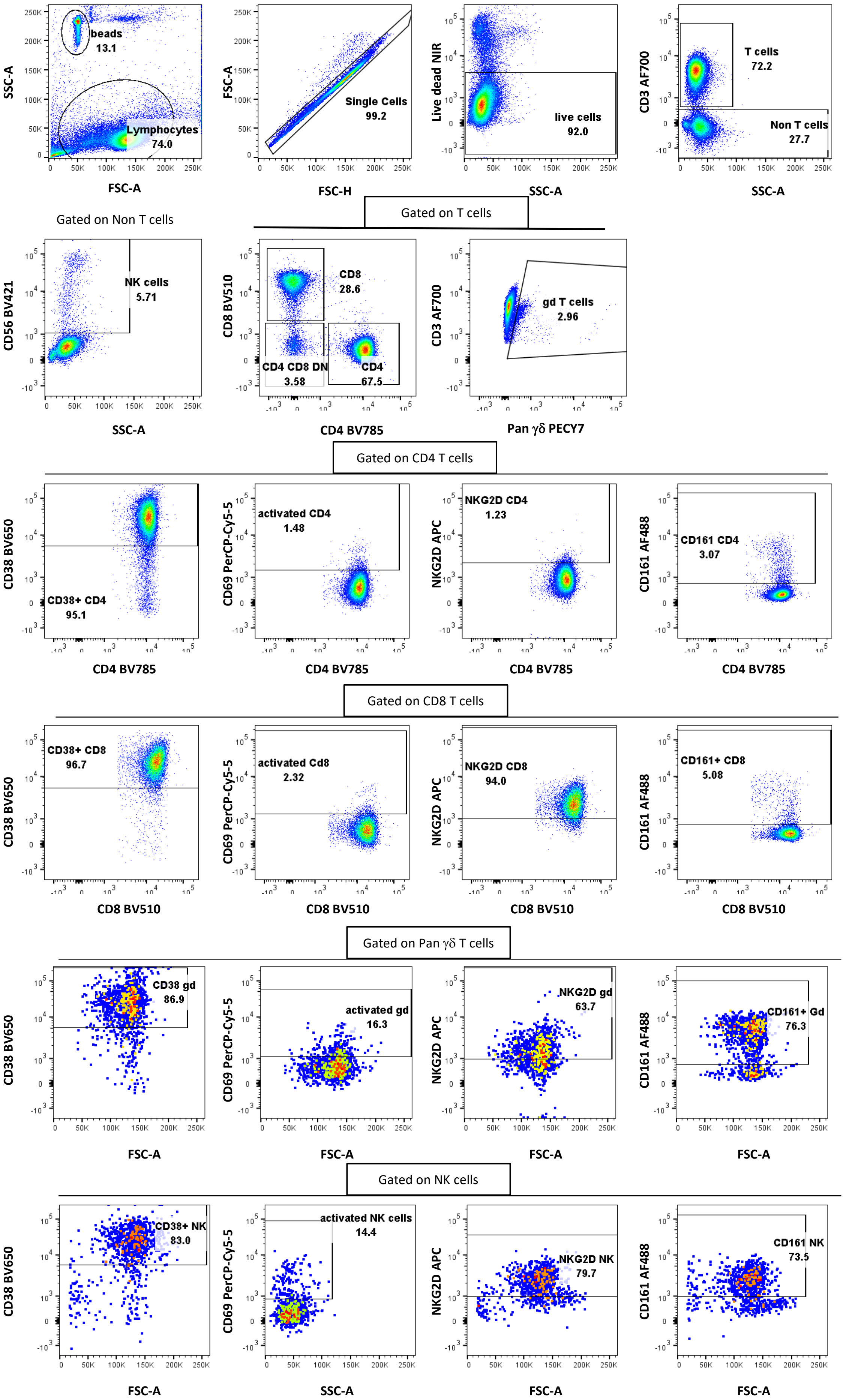

Supplementary figure 13: Gating strategy used to examine CD38, CD69, NKG2D and CD161 expression in NK cells, CD4, CD8 and γδ T cells by flow cytometry. This strategy was used to examine relevant parameters shown in figures 2,3 and 5 and supplementary figures 1,2, 6 and 7.

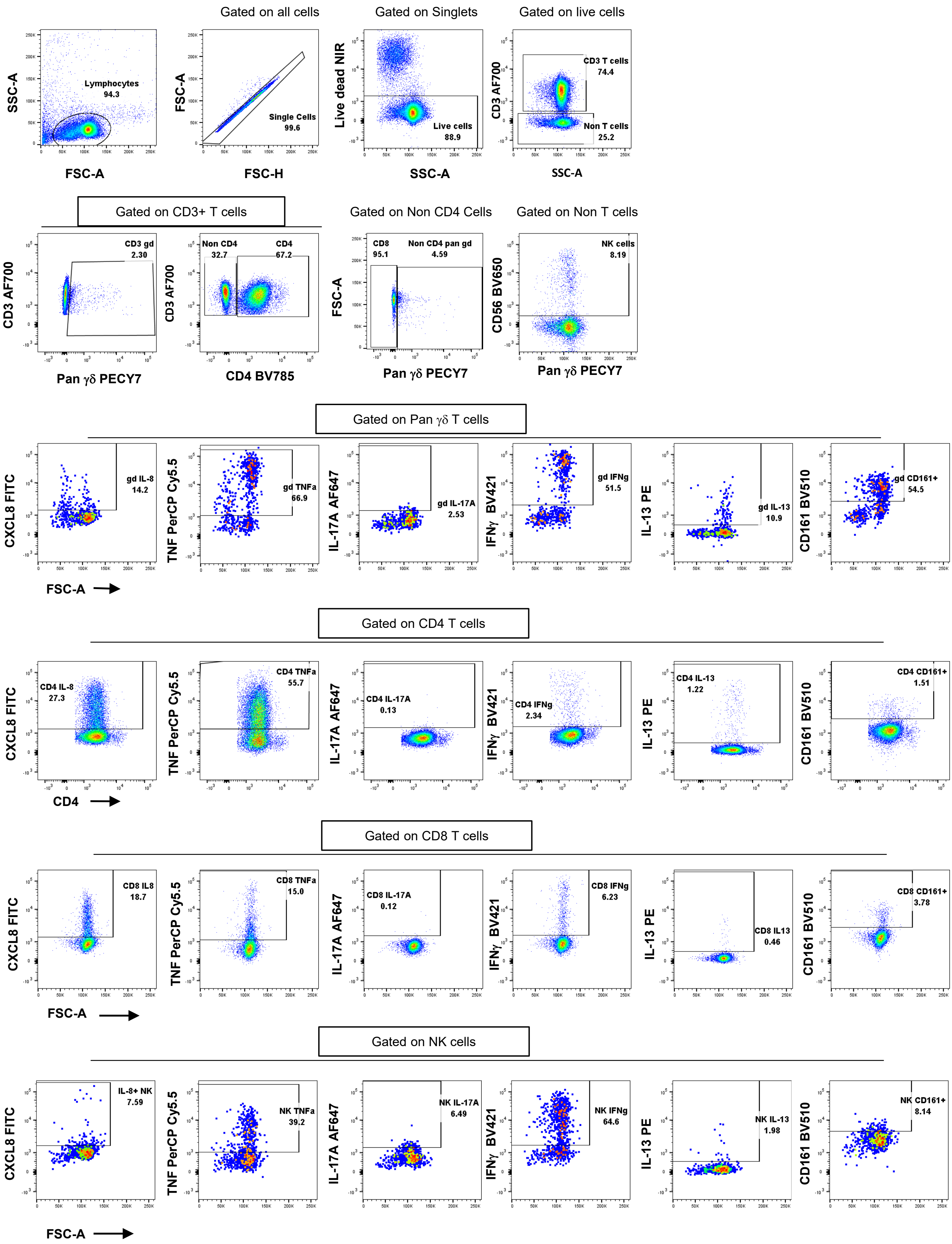

Supplementary figure 14: Gating strategy used for examining CXCL8, IFN-γ, TNF, IL-13 and CD161 in NK cells, CD4, CD8 and γδ T cells by flow cytometry. This strategy was used to examine relevant parameters shown in figures 2,3, 5 and 6 and supplementary figures 1,3,4,6 and 9.

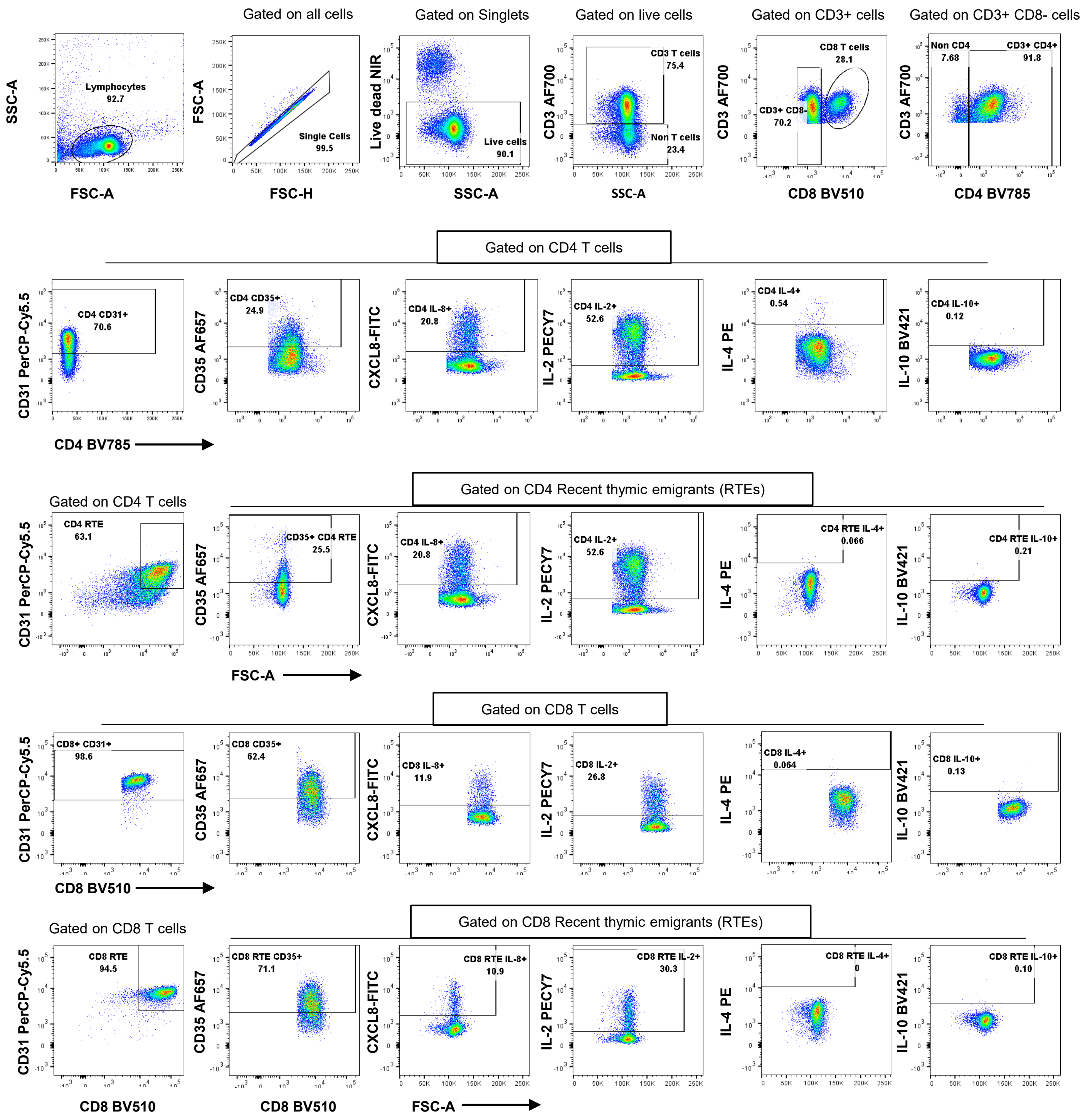

Supplementary figure 15: Gating strategy used for examining CD31, CD35, CXCL8, IL-2, IL-4 and IL-10 expression in CD4 and CD8 T cells and CD4 and CD8 recent thymic emigrant cells by flow cytometry. This strategy was used to examine IL-2 expression shown in figures 2,3,5 and 6 and supplementary figure 1,6 and 9, CD31 expression in figures 2, 3 and 5 and supplementary figures 1,6 and 7 and CD35 expression in figures 2, 3 and 5 and supplementary figures 1,6 and 8.

Supplemental Table 1: Antibodies used for flow cytometry

Marker	Fluorochrome	Company	Catalogue No.	Clone	Dilution
**Surface markers**					
Human Trustain Fcx	N/A	Biolegend	422302	N/A	2.5ul/test
CD3	AF700	Biolegend	317339	OKT3	1:200
CD4	PE-Cy7	Biolegend	317413	OKt4	1:50
CD4	BV786	Biolegend	317442	Okt4	1:50
CD8	BV510	Biolegend	301047	RPA-T8	1:400
Vδ1	FITC	Thermo Scientific	TCR2730	TS8.2	1:100
Vδ2	PerCP	Biolegend	331410	B6	1:50
CD45RA	BV786	Biolegend	304139	HI 100	1:50
CD45RA	BV650	Biolegend	304135	H100	1:250
CCR7	BV650	Biolegend	353233	GO43H7	1:50
CD95	BV421	Biolegend	305623	dx2	1:50
CD25	PE	Biolegend	356103	MA-251	1:50
CD27	PE	Biolegend	356103	MA-251	1:50
CD19	BV786	Biolegend	302239	HIB 19	1:100
CD14	BV421	Biolegend	301829	M5E2	1:100
CD16	APC/CY7	Biolegend	301819	3g8	1:100
CD14	APC/CY7	Biolegend	302017	M5E2	1:100
CD16	PE-Cy7	Biolegend	302015	3g8	1:200
HLADR	AF488	Biolegend	307619	L243	1:100
CD86	PE	Biolegend	305405	IT2.2	1:100
CD40	BV510	Biolegend	334329	5c3	1:100
CD1c(BDCA-1)	APC	Biolegend	331523	L161	1:100
CD56	BV650	Biolegend	362532	5.1h11	1:50
CD123	PerCP Cy5.5	Biolegend	306015	6H6	1:50

CD303	FITC	Biolegend	307619	L243	1:100
CD11c	BV650	Biolegend	301637	3.9	1:50
Pan γδ	PE-Cy7	Biolegend	331222	B1	1:50
NKG2D	APC	Biolegend	320807	1D11	1:50
CD56	BV650	Biolegend	362532	5.1h11	1:50
CD56	BV421	Biolegend	362551	5.1h11	1:200
CD69	PerCP-Cy5.5	Biolegend	310925	FN50	1:50
CD161	AF488	Biolegend	339923	HP-3G10	1:50
CD161	BV510	Biolegend	301047	HP-3G10	1:40
CD38	BV650	Biolegend	356619	HB-7	1:100
CD31	PerCP-Cy5.5	Biolegend	303131	WM59	1:400
CD35	AF647	BD	565329	E11	1:50
**Intracellular markers**					
Ki67	BV421	Biolegend	350505	Ki-67	1:50
IFNγ	BV421	Biolegend	502531	4S.B3	1:50
TNFα	PerCP-Cy5.5	Biolegend	502925	MAB11	1:50
IL17A	AF647	Biolegend	512309	BL168	1:50
IL-13	PE	Biolegend	501903	JES10-5A2	1:50
IL-8	FITC	Biolegend	511406	E8N1	1:50
IL-2	PE-CY7	Biolegend	500325	MQ1-17H12	1:50
IL-4	PE	Biolegend	500808	MP425d2	1:50
IL-10	BV421	Biolegend	501421	Jes3-97d	1:50
FoxP3	APC	Biolegend	320214	259 d	1:50
